# Diffusion Tensor Imaging Revealing the Relation of Age-Related Differences in the Corpus Callosum With Cognitive Style

**DOI:** 10.3389/fnhum.2020.00285

**Published:** 2020-07-17

**Authors:** Shulan Hsieh, Zai-Fu Yao, Meng-Heng Yang, Cheng-Ta Yang, Chun-Hao Wang

**Affiliations:** ^1^Department of Psychology, National Cheng Kung University, Tainan, Taiwan; ^2^Institute of Allied Health Sciences, National Cheng Kung University, Tainan, Taiwan; ^3^Department and Institute of Public Health, National Cheng Kung University, Tainan, Taiwan; ^4^Brain and Cognition, Department of Psychology, University of Amsterdam, Amsterdam, Netherlands; ^5^Institute of Physical Education, Health and Leisure Studies, National Cheng Kung University, Tainan, Taiwan

**Keywords:** analysis-holism scale, cognitive style, diffusion-weighted imaging, white matter tract, corpus callosum

## Abstract

People may differ in their ways of processing tasks or situations, which may be explained by cognitive styles that define individual differences in information processing strategies. The cognitive style ranges between two extremes: analytic and holistic processing style. The concept of cognitive style has been widely investigated in the literature, but its age-related differences in the neural substrates have remained elusive. In this study, we focused on the white matter structure of the corpus callosum and its possible link to age-related differences in cognitive style, given its functional ability to connect and facilitate efficient communication between the left and right cerebral hemispheres. Seventy-two participants aged 20–75 years participated in this study. Participants’ cognitive styles were measured by the Analysis-Holism Scale (AHS), and their white matter microstructures were acquired using diffusion-weighted magnetic resonance imaging. The results revealed that older adults tend to have a more holistic processing style than younger adults. We then compared the white matter of tracts of interest between high and low AHS groups and found that the white matter microstructure in the genu of the corpus callosum can be used to distinguish between AHS subgroups. Interestingly, we found that age negatively correlated with the white matter tracts across the brain, indicating that aging is associated with reduced microstructure integrity. Together, our findings suggest that analytic-holistic cognitive styles of information processing possibly reflect that the microstructure development in the anterior part of the corpus callosum may influence the type of age-related information processing.

## Introduction

### Cognitive Style and Its Constructs

Cognitive style refers to an individual’s attitudes, preferences, or strategies for acquiring and processing information, which involves particular cognitive functions such as perceiving, remembering, thinking, and problem-solving (Messick, 1984; Kozhevnikov, 2007; Mealor et al., 2016). The implications of cognitive style have been widely studied for decades across various research fields, including business, management, education, and psychology (Coffield et al., 2004). Various dimensional models and measurement methods (questionnaires or diagnostic tools) have been developed to define the cognitive style. Nevertheless, Miller (1987) suggested that even though there are various terms for cognitive style based on different models, most terms may be subordinate to and reflect a broad stylistic difference that represents a long-established distinction between two contrasting cognitive style constructs. The first cognitive style construct is commonly described using the terms “analytical,” “deductive,” “rigorous,” “constrained,” “convergent,” “formal,” and “critical.” The second cognitive style construct is commonly described using the terms “synthetic” (or “holistic”), “inductive,” “expansive,” “unconstrained,” “divergent,” “informal,” “diffuse,” and “creative” (Nickerson et al., 1985).

### “Hemispheric Preference” Theory

At the neural level, the two contrasting cognitive style constructs suggested by Miller (1987) have been linked to the distinction between ways of thinking based on the left and right hemispheres (Leonard and Straus, 1997). This linkage is based on the assumption that each hemisphere has different cognitive functions when processing information (Prevedi and Carli, 1987; Riding et al., 1993; Gazzaniga, 2005; Schulte and Müller-Oehring, 2010; Hinkley et al., 2012). It has been suggested that the left hemisphere involves rational, convergent, realistic, objective, and critical thinking. In contrast, the right hemisphere involves holistic, synthetic, intuitive, analogical, divergent, and creative thinking (Prevedi and Carli, 1987; Leonard and Straus, 1997).

In line with this idea, some studies (Müller-Oehring et al., 2009; Chechlacz et al., 2015; Nash et al., 2015) using global-local hierarchical structure figures have shown that global and local aspects of visual stimuli are differentially processed in the two hemispheres (Peters, 2004): local aspects are processed preferentially by the left hemisphere, whereas global aspects of stimuli are processed by the right hemisphere. Despite hemispheric lateralization for unilateral localization of function being found, the evidence to support the distinction between left- and right-hemisphere types of cognitive styles (e.g., analytic vs. holistic style) is still unsettled.

### Aging, Reduced Hemispheric Asymmetry, and the Corpus Callosum

As people age, the effect of hemispheric preference (i.e., lateralization) has been found to decrease (Müller-Oehring et al., 2007). For example, previous research using Navon (1977) global-local hierarchical stimulus paradigm has found that aging reduces hemispheric lateralization (Cabeza et al., 2002; Daselaar and Cabeza, 2005). Such a reduction in hemispheric lateralization has been further associated with the degradation of corpus callosum (Müller-Oehring et al., 2007).

Age-related thinning of the corpus callosum has been repeatedly reported (though still controversial). Studies involving older adults show age-related atrophy in the anterior and middle sections of the corpus callosum, while the posterior part does not appear to be susceptible to age-related atrophy (Salat et al., 1997; Persson et al., 2006; Junle et al., 2008). Studies applying diffusion tensor imaging (DTI) showed that age-related decline in callosal thinning appears to be robust and is correlated with slower reaction times in interhemispheric information processing or a transfer time task.

The effects of old age involve a significant thinning of the callosal volume according to MRI and post-mortem studies (Weis et al., 1993; Hopper et al., 1994; Sullivan et al., 2002). Abe et al. (2002) used DTI and found that the integrity of white matter tracts in the genu of the corpus callosum declined significantly with increasing age. Reduced callosal integrity was found to affect the speed of interhemispheric transfer time, which can occur during the natural aging process or as an effect of alcoholism, and the transfer time task affects both motor and sensory processes (Schulte et al., 2004).

### Cooperative vs. Inhibition Function of the Corpus Callosum

The functions of the corpus callosum concerning interhemispheric communication remain to be determined (Bloom and Hynd, 2005). The communication could occur using integrating motor, sensory, and cognitive performance between the cerebral cortex on one side of the brain with the same region on the other side through cooperative function (Banich and Belger, 1990; Banich et al., 1990). Alternatively, the communication might result from the inhibition of the other inferior side of information processing through inhibitory function (Chiarello and Maxfield, 1996).

Müller-Oehring et al. (2007) further suggest that the functions of the corpus callosum might involve both inhibition and cooperation depending on the part of the corpus callosum (the genu vs. the splenium). They designed a global-local hierarchical letter paradigm (e.g., a global letter of  “F” made out of local letters of “E”s) to test the hypothesis that aging reduces functional hemispheric lateralization through the degradation of the corpus callosum. Their results suggest that a reduced size of the corpus callosum (especially the genu in the anterior part) as a result of aging leads to less robust inhibition based on the inhibitory function of the corpus callosum. Thus, hemispheric lateralization is reduced, and interference with the processing of local and global letters is made possible (i.e., interference from local incongruent information and response inhibition from conflicting local information while processing global information). Conversely, in aging brains, the splenium in the posterior part of the corpus callosum appears to be associated with less facilitation function based on the cooperative function of the corpus callosum (Müller-Oehring et al., 2007).

### Aging and Cognitive Style

Compared to younger adults, previous studies have observed that older adults exhibit more holistic processing (Hsieh et al., 2020), context-dependent processing (Demick and Wapner, 1991), intuitive processing (Racine et al., 2006), external control (Lachman, 2006), and integration styles (Radvansky, 1999; Dror et al., 2005). Moreover, Chan et al. (2018) also found that older adults exhibited more holistic patterns during facial expression recognition than younger adults (Chan et al., 2018). The evidence from these studies converges to suggest that older adults tend to adopt a holistic cognitive style (Pask, 1972). Additional studies using a global-local stimulus have found that local processing declines with age, whereas global processing is preserved with age (Roux and Ceccaldi, 2001; Meng et al., 2019).

### Aims of the Study

Given that aging degrades the corpus callosum while it enhances holistic-processing tendencies, it is interesting to explore whether these two phenomena are related. Therefore, this study aims to examine the complex relationships among age, holistic-analytic style, and the communication efficacy of the corpus callosum. We specifically focused on the communication of the corpus callosum reflected in forceps minor (Fmin) and forceps major (Fmaj) in the DTI measures, in which Fmin represents the genu while Fmaj represents the splenium of the corpus callosum. Nevertheless, since there’s no prior research reported the complex relationship among age, DTI, and cognitive style, we in parallel explored the whole brain white matter tracts in order not to overlook other white matter tracts’ potential role in the relationship. To achieve this goal, we evaluated analytic vs. holistic thinking styles using the Analysis-Holism Scale (AHS) developed by Choi et al. (2007). The AHS has been translated into a Chinese version by Jen and Lien (2010). The communication efficacy of the corpus callosum and other white matter tracts was evaluated using Diffusion-weighted imaging (DWI) data fitted by the diffusion tensor model (Horsfield and Jones, 2002; Alexander et al., 2007; Soares et al., 2013).

Common measures of DTI include fractional anisotropy (FA) and the mean and radial diffusivity (MD and RD, respectively). The FA value reflects the fiber density, axonal diameter, and myelination in the white matter, while MD provides information about the overall omnidirectional diffusion. RD is defined as the magnitude of water *diffusion* perpendicular to the tract and is generally considered a sensitive measure for axonal/myelin damage (Wheeler-Kingshott and Cercignani, 2009).

To the best of our knowledge, there has been no prior research directly examining the relationship between AHS scores and DTI across the adult lifespan. Despite this, we could generate two testable hypotheses based on the cooperative vs. inhibition hypotheses of the corpus callosum in conjunction with the hemispheric preference theory in the literature. According to the cooperative hypothesis of the corpus callosum, we might expect to observe that the FA in the corpus callosum would decrease (or MD and/or RD might be increased) as a function of AHS scores, because the lower transmission between the two hemispheres would paradoxically enhance the hemispheric advantage of the processing, such as right-hemisphere biased in processing the global information (i.e., more holistic). In contrast, according to the inhibition hypothesis of the corpus callosum, one would predict that FA might increase (or MD and/or RD might be decreased) as a function of AHS scores because a higher level of interhemispheric inhibition would paradoxically maintain the original right-hemispheric biased processing.

## Materials and Methods

### Participants

A total of 80 participants (38 females and 42 males) were recruited for the present study. The criteria for recruitment included right-handedness according to the Edinburgh Handedness Inventory (Oldfield, 1971), no major neurological or psychological disorders based on the participants’ self-declaration, and normal or corrected-to-normal vision. Also, participants were excluded from the data analysis if they had scores <22 (*n* = 1) on the Montreal Cognitive Assessment (MoCA; screening for probable dementia; Nasreddine et al., 2005) or scores > 13 (*n* = 7) on the Beck Depression Inventory-II (BDI-II; screening for depression; Beck et al., 1996).

Table 1 shows the demographic information of the 72 participants that met the criteria, including scores on neuropsychological batteries. All participants signed an informed consent form before participating in the experiment (including consent for MRI acquisition and cognitive style assessment by the AHS). All subjects were paid 1,500 NTD (around USD 50) after completion of the experiment. The study protocol was approved by the Institutional Review Board of the Human Research Ethics Committee of National Cheng Kung University, Tainan, Taiwan. The total time to complete the AHS was around 15 min, and the total time to complete all MRI scanning sessions was within 60 min.

**Table 1:** Demographic information and scores on neuropsychological batteries for 72 participants.

*n* = 72	Mean ± SD	Range
Age (years)	50.12 ± 20.00	20.75–75.43
Education (years)	14.29 ± 2.87	6–18
MoCA	28.17 ± 1.78	23–30
BDI-II	3.72 ± 3.58	0–13
AHS	131.51 ± 17.85	79–167

*MoCA, Montreal Cognitive Assessment; BDI-II, Beck Depression Inventory-II; AHS, Analysis-Holism Scale*.

### Cognitive Style Assessment

The AHS was used to assess an individual’s analytic vs. holistic thinking style (Choi et al., 2007; Chinese version by Jen and Lien, 2010). The AHS is a self-reported questionnaire with 24 items and four subscales (causality, attitude toward contradictions, perception of change, and locus of attention). Each item is scored on a seven-point Likert-type scale [from 1 (strongly disagree) to 7 (strongly agree)]. The AHS score is the sum of the 24 items’ points. Higher scores indicate a more holistic thinking style, and lower scores indicate a more analytic thinking style.

### Image Acquisition and Processing

MRI data were acquired on a GE MR750 3T scanner (GE MR750 Healthcare, Waukesha, WI, USA) located in the Mind Research Imaging Center at National Cheng Kung University. High-resolution structural images were acquired with the Fast Spoiled Gradient Echo (Fast SPGR) sequence (166 axial slices; repetition time (TR) = 7.6 ms; echo time (TE) = 3.3 ms; flip angle = 12°; field of view (FOV) = 22.4 × 22.4 cm^2^; matrices = 224 × 224; slice thickness = 1 mm; total time = 218 s). DWI was acquired with a gradient-echo echo-planar sequence (TR/TE = 5,500 ms/62–64 ms, 50 directions with *b* = 1,000 s/mm^2^, 100 × 100 matrices, slice thickness = 2.5 mm, voxel size = 2.5 × 2.5 × 2.5 mm, number of slices = 50, FOV = 25 cm, NEX = 3). Reverse DWI was also acquired for top-up correction in the DWI preprocessing. The acquisition parameters for the reverse DWI were identical to those of the DWI; the only difference was that the number of directions was changed to six.

### DWI Preprocessing

We first converted raw DWI images from the DICOM format to the NIFTI format using MRIcron’s dcm2nii tool^1^. We then preprocessed all DWI data using the FMRIB Software Library (FSL v5.0.9^2^, Smith et al., 2004; Jenkinson et al., 2012). For each participant and session, DWI data were concatenated and corrected for eddy currents (EDDY in FSL) following top-up correction (TOP-UP in FSL) to reduce artifacts caused by susceptibility-induced distortions, eddy currents, and head motion. Visually inspection of each acquired volume was done by checking outputs parameters from correction steps (i.e., EDDY and TOPUP) after correction for signal loss, distortion, Venetian blind, bar artifact, and other anomalies.

Affine registration was then used to register each volume to a reference volume (Jenkinson and Smith, 2001). A single image without diffusion weighting (b0; *b* value = 0 s/mm^2^) was extracted from the concatenated data, and non-brain tissue was removed using the FMRIB Brain Extraction Tool (BET; Smith, 2002) to create a brain mask for subsequent analyses. Finally, the DTI fitting model (DTIFIT; Smith et al., 2004) was applied to fit a tensor model at each voxel of the data to derive FA, MD, and RD measures for further analyses.

### Tract-Based Spatial Statistics

After preprocessing, we used whole-brain tract-based spatial statistics (TBSS, Smith et al., 2006) in FSL to enable tract-based investigations of the DTI measures, which involves voxel-wise statistical analyses of the DWI data. We used the following procedure to examine the quality of each measure for each diffusion measure. First, FA images were slightly eroded, and end slices were zeroed to remove likely outliers from the diffusion tensor fitting. The images were then nonlinearly aligned (i.e., tbss_2reg_ in FSL) to the FMRIB58_FA_ standard-space image. This target image was subsequently affine-transformed to 1 mm MNI space.

Next, each participant’s FA images were transformed to a 1-mm MNI space by combining the nonlinear and affine registration. A skeletonization procedure was then performed on the group-mean FA image, and the result was thresholded at FA > 0.2 to identify areas most likely to belong to white matter tracts. This process was then repeated for MD and RD images using the tbss_non_FA function.

### White Matter: Fiber Tracts Processing

For tract-of-interest analyses, TBSS skeletoned FA/MD/RD maps were overlaid with atlas binary masks based on the probabilistic John Hopkins University (JHU) white-matter tractography atlas (ICBM-DTI-81) in *FSL* (provided by the ICBM DTI workgroup), which were created with a probability threshold of 5%. The values of each diffusion scalar were computed for these tracts for each participant. Eighteen tracts were targeted in this study, including anterior thalamic radiation (ATR), cingulum (CG), cingulum of the hippocampus (CH), corticospinal tract (CST), inferior fronto-occipital fasciculus (IFF), inferior longitudinal fasciculus (ILF), superior longitudinal fasciculus (SLF), and uncinate fasciculus (UF) in left and right hemispheres, respectively, as well as Fmaj, and Fmin. Particularly, primary tracts of interest in Fmaj and Fmin are parts of the corpus callosum. Fmin is the commissural fibers of the anterior corpus callosum cross over and give rise to a connection between regions of the frontal cortices. Whereas Fmaj is the commissural fibers of the posterior corpus callosum cross over and gives rise to a connection between regions of the occipital lobes.

### Statistical Analyses

Linear regressions were first performed in SPSS v22 (IBM, Armonk, NY, USA) to test for partial linear association between the participants’ age and their self-reported AHS scores while controlling for gender, education, and MoCA, and BDI-II scores. The second set of linear regressions was used to test for the partial linear association between DTI measures (FA, MD, and RD) and age while controlling for gender, education, and scores of MoCA and BDI-II (1 regression per measure). Lastly, we tested for the partial linear association between DTI measures (FA, MD, and RD) and AHS scores, again controlling for gender, education, and scores of MoCA and BDI-II (1 regression per measure). Furthermore, we used the JASP (Version 0.11.0.0) to perform the Bayesian version of statistical tests. Specifically, the Bayes factor provides an easily interpretable index of preference for one hypothesis over another that has two primary advantages over traditional null hypothesis testing techniques. First, it provides a direct measure of evidence, which we define as the extent to which a set of observed data should update our belief in one hypothesis over the other. Second, whereas traditional null hypothesis testing does not allow one to “accept” a null hypothesis, it is perfectly acceptable and well-defined to measure the evidence in favor of a null hypothesis by computing a Bayes factor. All of these partial linear regression procedures were applied for the DTI analyses of the whole brain and tracts of interest.

For the voxel-wise univariate regressions, we used FSL’s randomize function (a nonparametric permutation method for MRI data^3^) on a model mimicking the whole-brain average regressions while testing for the linear association between age and DTI measures (FA, MD, and RD) and controlling for gender, education, and scores of MoCA and BDI-II. We also performed voxel-wise univariate regressions for the DTI measures and AHS scores, again controlling for gender, education, and scores of MoCA and BDI-II.

For both the behavioral and DTI results, we report classical frequentist *p*-values and Bayes factors, which provide a more conservative evaluation of the correlations. We provide both BF_10_ (Bayes factor for the presence of a correlation) and BF_01_ (Bayes factor for the absence of a correlation) for ease of reading. These factors are inversely related (BF_10_ = 1/BF_01_ and BF_01_ = 1/BF_10_). The Bayes factors may be interpreted as proportional evidence for the presence or absence of an effect. For instance, a BF_10_ of five indicates that the data are five times more likely to occur under the alternative hypothesis than under the null-hypothesis.

Also, the between-group *t*-test comparison analyses were conducted. We divided the participants into two subgroups based on their AHS scores by the median split. We then compared the DTI measures of the whole brain and tract of interest between the two subgroups by using the Bayesian independent *t*-tests with the Bayes factor.

## Results

### Descriptive Data of Cognitive Style

The mean ± standard deviation (SD) of the AHS score was 131.51 ± 17.85 with a range of 79–167 (Table 1).

### Age and AHS

**Figure 1:**
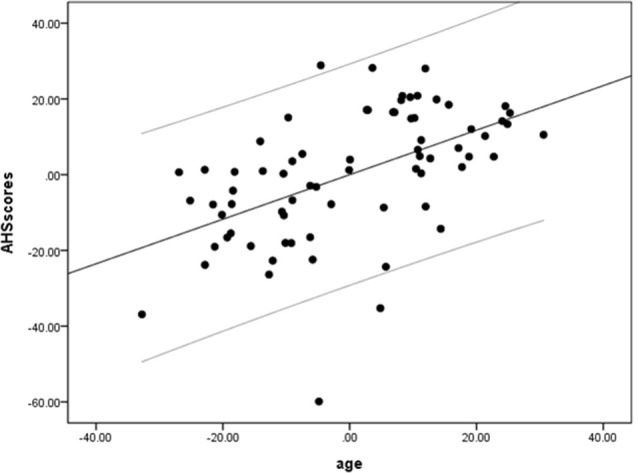
A positive correlation between age and Analysis-Holism Scale (AHS) scores. Scatterplot with regression line partially regressing out variance attributed to gender, education, and scores of Montreal Cognitive Assessment (MoCA) and Beck Depression Inventory-II (BDI-II).

We performed a partial linear regression analysis predicting AHS scores based on age while controlling for gender, education, MoCA, and BDI-II scores. The result showed a significant correlation of AHS scores with age (*r* = 0.66, *p* = 0.000004, BF_10_ = 3612.56; Figure 1), suggesting that older adults have higher holistic tendencies than younger adults.

### Age and DTI

**Figure 2:**
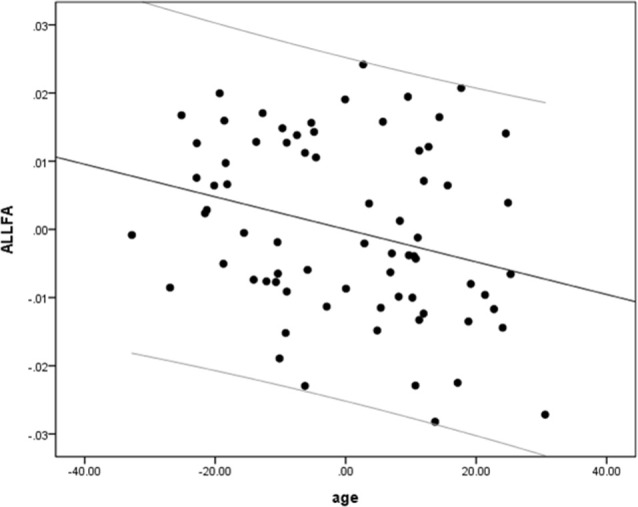
A negative correlation between age and fractional anisotropy (FA). Scatterplot with regression line between age and FA regressing out variance attributed to gender, education, and scores of MoCA and BDI-II.

**Figure 3:**
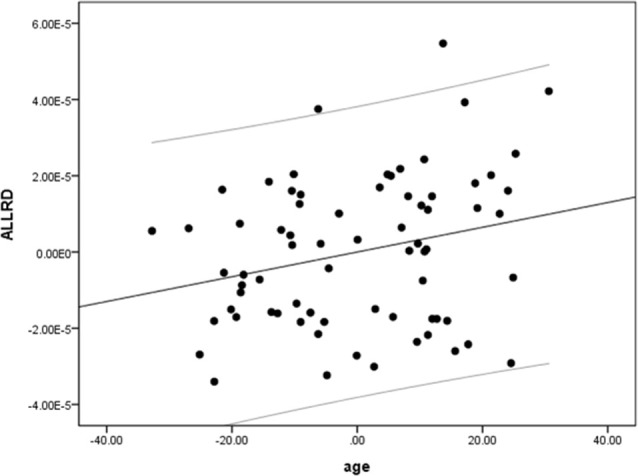
A positive correlation between age and radial diffusivity (RD). Scatterplot with regression line between age and RD regressing out variance attributed to gender, education, and scores of MoCA and BDI-II.

We performed a partial linear regression for predicting whole-brain average DTI measures (FA, MD, and RD) based on age while regressing out variance attributed to gender, education, and scores of MoCA and BDI-II. We observed a negative correlation between FA and age (*r* = −0.36, *p* = 0.02, BF_10_ = 4.03; Table 2 and Figure 2). We also extracted whole-brain skeleton-averaged MD and RD and regressed these against the participants’ age (again controlling for gender, education, and scores of MoCA and BDI-II; see Table 2). No significant correlations were found between age and MD, but a significant positive correlation was found between age and RD (*r* = 0.32, *p* = 0.04, BF_10_ = 2.67; see Table 2 and Figure 3).

**Table 2:** Mean with standard deviation (SD) of FA/MD/RD and their correlation results with age (partially regressing out variance attributed to gender, education, MoCA, and BDI-II).

	Mean ± SD	*r*	*p*	BF_10_	BF_01_
FA	0.437 ± 0.013	−0.36	0.02	4.03	0.25
MD	(0.766 ± 0.018)*10^−3^	0.09	0.57	0.54	1.85
RD	(0.565 ± 0.020)*10^−3^	0.32	0.04	2.67	0.37

*FA, fractional anisotropy; MD, mean diffusivity; RD, radial diffusivity*.

### Age and DTI Voxel-Based Regression

**Figure 4:**
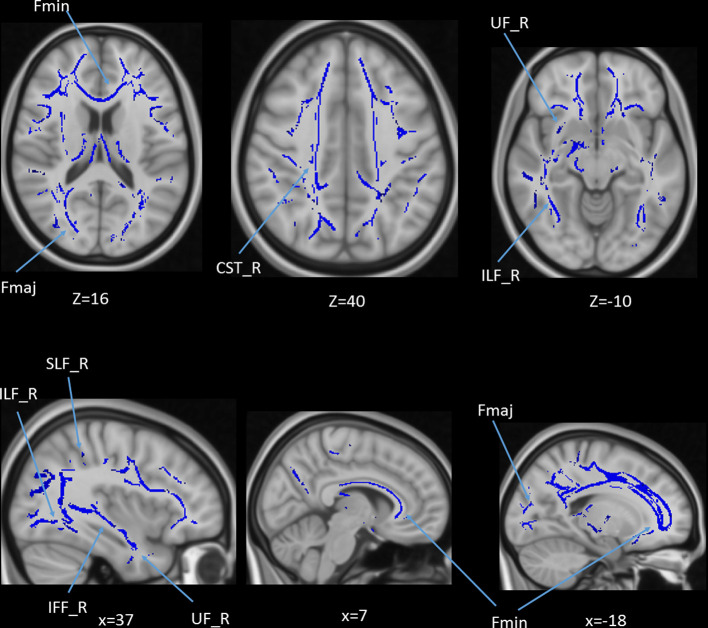
Statistical map showing white matter clusters (blue) where FA was significantly negatively correlated with age (controlling for gender, education, and scores of MoCA and BDI-II). Axial slices are presented in radiological orientation (right = left). CST, corticospinal tract; Fmaj, forceps major; Fmin, forceps minor; IFF, inferior fronto-occipital fasciculus; ILF, inferior longitudinal fasciculus; SLF, superior longitudinal fasciculus; UF, uncinate fasciculus.

**Figure 5:**
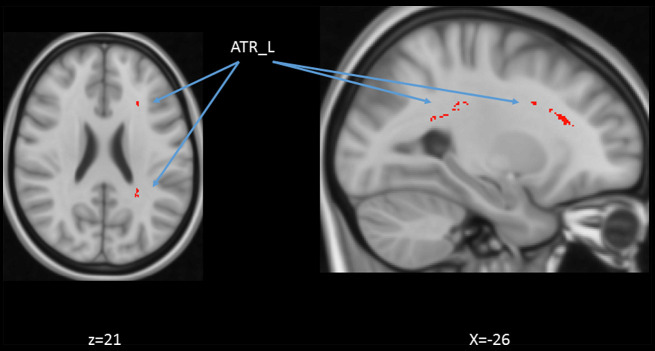
Statistical map showing white matter clusters (red-yellow) where mean diffusivity (MD) was significantly positively correlated with age (controlling for gender, education, and scores of MoCA and BDI-II). Axial slices are presented in radiological orientation (right = left). ATR: anterior thalamic radiation.

**Figure 6:**
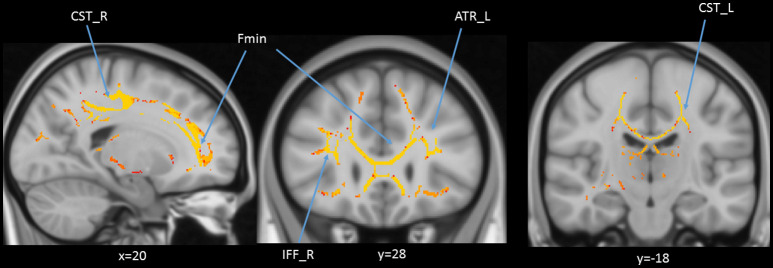
Statistical map showing white matter clusters (red-yellow) where radial diffusivity (RD) was significantly positively correlated with age (controlling for gender, education, and scores of MoCA and BDI-II). Axial slices are presented in radiological orientation (right = left). ATR, anterior thalamic radiation; CST, corticospinal tract; Fmin, forceps minor; IFF, inferior fronto-occipital fasciculus.

We also performed a mass-univariate regression analysis (again controlling for gender, education, and scores of MoCA and BDI-II) within the whole-brain white matter skeleton to pinpoint spatially localized relations between age and white matter microstructure (i.e., voxel-wise statistical analyses). We found negative relations between age and FA in clusters of 11 tracts (Figure 4), positive correlations between age and MD in clusters of one tract (Figure 5), and positive correlations between age and RD in clusters of five tracts (Figure 6).

### Age and DTI Tract-of-Interest Analyses

**Figure 7:**
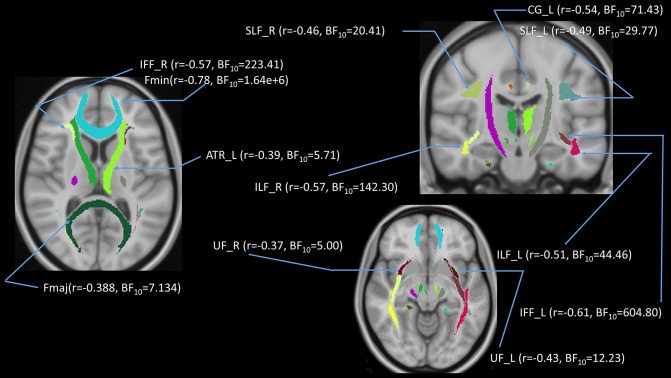
Partial correlations (controlling for gender, education, and scores of MoCA and BDI-II) between age and FA in 12 out of 18 *white matter tracts*. Note: r = partial correlation value, BF_10_ = Bayes factor for the presence of a correlation; L = left hemisphere; R = right hemisphere. ATR, anterior thalamic radiation; CG, cingulum; Fmaj, forceps major; Fmin, forceps minor; IFF, inferior fronto-occipital fasciculus; ILF, inferior longitudinal fasciculus; SLF, superior longitudinal fasciculus; UF, uncinate fasciculus. Note: colors in the figure represent the different tracts of interest.

**Figure 8:**
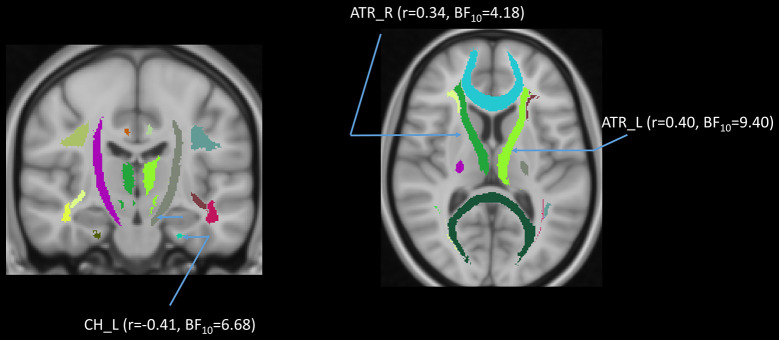
Partial correlations (controlling for gender, education, and scores of MoCA and BDI-II) between age and mean diffusivity (MD) in 3 out of 18 *white matter tracts*. Note: r = partial correlation value, BF_10_ = Bayes factor for the presence of a correlation; L = left hemisphere; R = right hemisphere. ATR, anterior thalamic radiation; CH, cingulum of the hippocampus. Note: colors in the figure represent the different tracts of interest.

**Figure 9:**
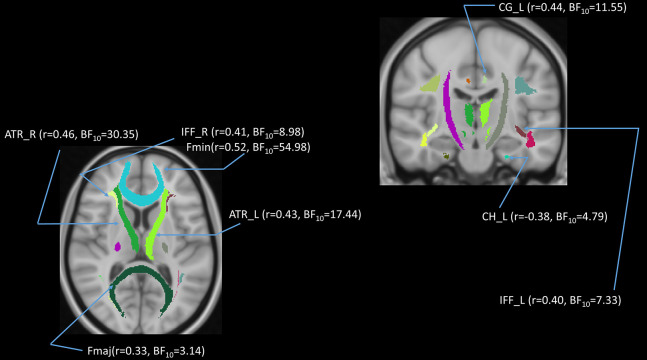
Partial correlations (controlling for gender, education, and scores of MoCA and BDI-II) between age and radial diffusivity (RD) in 8 out of 18 *white matter tracts*. Note: r = partial correlation value, BF_10_ = Bayes factor for the presence of a correlation; L = left hemisphere; R = right hemisphere. ATR, anterior thalamic radiation; CG, cingulum; CH, cingulum of the hippocampus; Fmaj, forceps major; Fmin, forceps minor; IFF, inferior fronto-occipital fasciculus; ILF, inferior longitudinal fasciculus. Note: colors in the figure represent the different tracts of interest.

In addition to the whole-brain DTI analyses, we were also interested in exploring which white matter tracts on the skeletoned FA, MD, and RD maps might be correlated with age. We performed tract-of-interest analyses and then partially correlated these tracts’ FA with age (controlling for gender, education, and scores of MoCA and BDI-II). The results showed that 12 out of 18 tracts were significantly negatively correlated with age (Figure 7). Most of the correlations were negative, suggesting the majority of the tracts’ FA decreased with age. The same procedures were conducted on MD and RD in the 18 tracts as well. The results showed that 3 out of 18 tracts in the case of MD and 8 out of 18 tracts in the case of RD were significantly correlated with age (Figures 8, 9). All these significant correlations achieved a Bayesian factor BF_10_ > 3.

**Figure 10:**
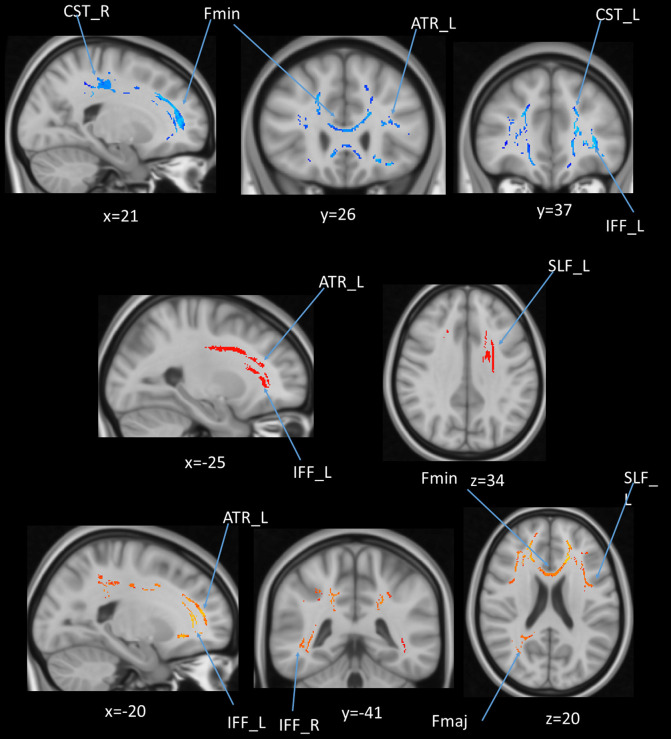
(upper panel): Statistical map showing white matter clusters (blue) where FA was significantly negatively correlated with AHS; (middle panel): statistical map showing white matter clusters (red-yellow) where mean diffusivity (MD) was significantly positively correlated with AHS; (lower panel): statistical map showing white matter clusters (red-yellow) where radial diffusivity (RD) was significantly positively correlated with AHS. Axial slices are presented in radiological orientation (right = left). ATR, anterior thalamic radiation; CST, corticospinal tract; Fmin, forceps minor; Fmaj, forceps major; Fmin, forceps minor; IFF, inferior fronto-occipital fasciculus; SLF, superior longitudinal fasciculus.

### DTI and AHS

In addition to cross-sectional age differences, we were interested in cross-sectional differences in DTI measures relating to AHS scores. We performed a linear regression between DTI measures and AHS while regressing out the gender, education, and scores of MoCA and BDI-II. We found no significant relation between AHS and FA (*r* = −0.14, *p* = 0.26, BF_01_ = 1.24). We subsequently investigated the remaining DTI measures (MD and RD), which were also found not to be associated with AHS scores (Table 3).

**Table 3:** Statistical results for the partial correlations (controlling for gender, education, and scores on MoCA and BDI-II) between DTI measures [fractional anisotropy (FA), mean diffusivity (MD), and radial diffusivity (RD)] and the Analysis-Holism Scale (AHS).

	*r*	*p*	BF_10_	BF_01_
FA	−0.14	0.26	0.81	1.24
MD	0.17	0.18	0.96	1.04
RD	0.22	0.07	1.70	0.59

### Voxel-Based Regression

We also performed a mass-univariate regression analysis on the whole-brain white matter skeleton to pinpoint spatially localized relations between AHS scores and white matter microstructure (i.e., voxel-wise statistical analyses). We found significant correlations between AHS and DTI tracts in FA (Fmin, CST in both hemispheres, ATR in the left hemisphere, and IFF in the left hemisphere; Figure 10 upper panels), MD (ATR in the left hemisphere, SLF in the left hemisphere, and IFF in the left hemisphere; Figure 10 middle panels), and RD measures (Fmin, Fmaj, and IFF in both hemispheres, ATR in the left hemisphere, and SLF in the left hemisphere; Figure 10 lower panel).

### AHS and DTI of Tracts of Interest

We performed tract-of-interest analyses and then partially correlated these tracts’ DTI measures with AHS. We found a significantly negative correlation between AHS and FA in the Fmin (*r* = −0.36, *p* = 0.002, BF_10_ = 21.26), IFF in both hemispheres (left: *r* = −0.29, *p* = 0.02, BF_10_ = 4.55; right: *r* = −0.28, *p* = 0.02, BF_10_ = 3.87), and ILF in both hemispheres (left: *r* = −0.28, *p* = 0.03, BF_10_ = 3.19; right: *r* = −0.29, *p* = 0.02, BF_10_ = 3.81). There were significantly positive correlations between AHS and MD in ATR in both hemispheres (left: *r* = 0.31, *p* = 0.01, BF_10_ = 8.99; right: *r* = 0.33, *p* = 0.01, BF_10_ = 10.96), as well as between AHS and RD in ATR in both hemispheres (left: *r* = 0.33, *p* = 0.01, BF_10_ = 11.89; right: *r* = 0.34, *p* = 0.00, BF_10_ = 12.99), Fmin (*r* = 0.32, *p* = 0.01, BF_10_ = 7.37), and IFF in both hemispheres (left: *r* = 0.28, *p* = 0.02, BF_10_ = 3.55; right: *r* = 0.28, *p* = 0.02, BF_10_ = 3.87).

### High vs. Low AHS Groups Comparison

**Figure 11:**
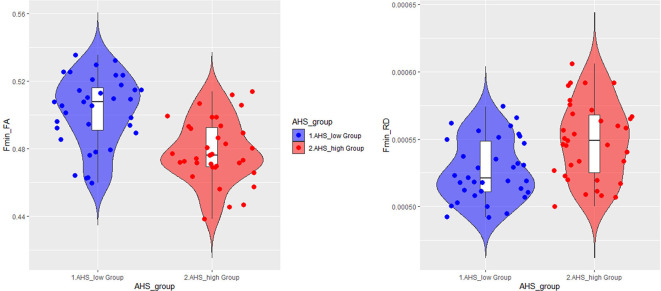
Violin plots for the high and low-AHS subgroups in FA (left panel) and RD (right panel) in the forceps minor (Fmin) tract. FA, fractional anisotropy; RD, radial diffusivity.

In addition to the regression analyses, we also directly compared two subgroups of participants based on their AHS scores using the median-split method (i.e., AHS = 133). The results in Table 4 show that there were significant differences between the two subgroups in age (low: 37.00 ± 19.30 years; high: 62.64 ± 10.33 years; BF_10_ = 4.410e+6), AHS scores (low: 116.60 ± 11.71; high: 146.34 ± 8.51; BF_10_ = 3.109e+15), FA in Fmin (BF_10_ = 3771.35), and RD in Fmin (BF_10_ = 40.99). The results showed that the major differences between the high and low-AHS subgroups in FA and RD are specifically in the Fmin tract (Figure 11), thus highlighting the role of the genu of the corpus callosum in the analytic-holistic cognitive style.

**Table 4:** Demographic information between the two subgroups based on their AHS scores (median = 133).

	Low AHS (<133) *n* = 35	High AHS (>133) *n* = 35	BF_10_
Age (years)	37.00 ± 19.30	62.64 ± 10.33	4.410e+6
Education (years)	14.91 ± 3.05	13.63 ± 2.54	1.16
MoCA	28.43 ± 1.67	28.00 ± 1.78	0.39
BDI-II	4.17 ± 3.85	3.34 ± 3.38	0.36
AHS	116.60 ± 11.71	146.34 ± 8.51	3.11e+15
Fmin-FA	0.50 ± 0.02	0.48 ± 0.02	3771.35
Fmin-MD	(0.77 ± 0.02)*10^−3^	(0.78 ± 0.03)*10^−3^	0.82
Fmin-RD	(0.53 ± 0.02)*10^−3^	(0.55 ± 0.03)*10^−3^	40.99

*MoCA, Montreal Cognitive Assessment; BDI-II, Beck Depression Inventory-II; AHS, Analysis-Holism Scale*.

## Discussion

We have investigated the complex relationships among the microstructure characteristics of white matter, age, and AHS. The results suggested a positive association between age and AHS scores, indicating that older adults tend to adopt a more holistic information-processing strategy than younger adults. As successfully replicated previous studies, that the age effect is negatively correlated with diffusion imaging measures in several white matter tracts across the brain, indicating that the reduced microstructure integrity advances with age.

The group comparison between high and low-AHS subgroups in terms of diffusion measures of specific white matter tracts showed that the differences in both FA and RD inside the genu of the corpus callosum may mostly account for the individual difference in AHS scores. Together, our results suggested that analytic-holistic cognitive styles possibly reflect the microstructure development in the anterior part of the corpus callosum, which influences the way of information processing. Below, we discuss the paired relationships among age, AHS, and DTI.

### Age and AHS

An attempt was made to replicate the previous observation of age-related AHS score changes with more stringent conditions after controlling for gender, education, MoCA, and BDI-II scores. We observed a positive correlation between age and AHS, suggesting that older adults exhibited a higher level of holistic thinking style. This result is consistent with previous studies showing an age-related increased tendency toward holistic thinking (e.g., Zhang et al., 2014; Hsieh et al., 2020). Moreover, a recent study using global-local paradigms found that aging impairs local geometrical perception while preserving global topological perception (Meng et al., 2019). Also, Roux and Ceccaldi (2001) also found that in a selective-attention task using Navon’s (1977) figure (local-global hierarchical stimuli), a complete global precedence effect was found for younger and older participants. However, for older adults, the global interference in local identification was more pronounced than for younger adults.

We specifically examined whether age differences in AHS are related to differences in brain structures, especially white matter structures. This hypothesis is motivated by previous research indicating that the analytic vs. holistic cognitive style may be related to the distinction between left- and right-hemisphere ways of thinking (Leonard and Straus, 1997). These styles are sensitive to aging due to atrophy in the corpus callosum (Salat et al., 1997; Persson et al., 2006; Junle et al., 2008).

### Age and DTI

We applied the DTI approach to examine whether age might deteriorate white matter structures, this examination is to replicate findings of age-related white matter structure declines. The results indeed showed a negative correlation between age and FA, suggesting that the diffusion tensor is less fractionally anisotropic in older individuals (i.e., less efficacy in fiber communication) than in younger individuals. The results are consistent with previous findings showing a general decline in FA with age (Salat et al., 1997; Vernooij et al., 2008; Sullivan et al., 2010; Jolly et al., 2016).

In addition to the whole-brain DTI analyses, we also examined tract-of-interest and whole-brain DTI using voxel-wise (randomization) analyses concerning age. The results of the FA measure showed that the majority of the tracts were negatively correlated with age, including some commissural fibers (e.g., the Fmin and Fmaj) and association fibers (e.g., the cingulate gyrus, superior longitudinal fasciculus, inferior longitudinal fasciculus, and uncinated fasciculus). In contrast, MD and RD showed fewer tracts related to age than FA. Similar results can be found in the voxel-based analyses showing that FA in clusters of many tracts decreased with age, along with MD in the ATR tract, and RD in clusters in a few tracts (mainly ATR, CST, and Fmin).

The results showed that FA plays a more important role than MD or RD in reflecting the age effect. Decreases in FA may arise from both a relative increase in RD and a relative increase in axial diffusivity. Also, MD provides information regarding the overall omnidirectional diffusion. FA and RD were inversely related to age, especially in terms of Fmin of the corpus callosum) i.e., when the negative FA-age correlation emerged in Fmin, RD was found to be increased as well). Thus, we may reason that with age, there is a general increase of omnidirectional diffusion, which favors radial diffusivity (two minor directions of diffusion) over axial diffusivity, which may be related to a decrease in FA in Fmin (Boekel and Hsieh, 2018).

### AHS and DTI

Our main interest was whether DTI differences with age are related to the increased holistic cognitive style with age, as reflected in AHS scores. We evaluated the relationship between the AHS and DTI measures with a focus on the tract-of-interest and voxel-wise randomization analyses to evaluate the function of the corpus callosum (e.g., Fmin and Fmaj tracts) concerning analytic-holistic cognitive style. Generally, we did not observe significant correlations between AHS and whole-brain DTI measures (FA, MD, and RD), but we did observe significant correlations between AHS and the FA measure in specific tracts (Fmin, IFF in both hemispheres, and ILF in both hemispheres), between AHS and MD measures in ATR (both hemispheres), and between AHS and RD in ATR (both hemispheres), Fmin, and IFF (both hemispheres). Whole-brain voxel-wise randomized analyses also showed similar results in that AHS showed significant correlations with FA in Fmin, ATR (left hemisphere), CST (both hemispheres), and IFF (left hemisphere), with MD in ATR (left hemisphere), IFF (left hemisphere), and SLF (left hemisphere), and RD in Fmin, Fmaj, ATR (left hemisphere), IFF (both hemispheres), and SLF (left hemisphere). Among these tracts, Fmin, IFF, and ATR tracts seem to play important roles concerning the AHS scores. These tracts are also closely associated with the anterior part of the corpus callosum, as the fiber projections from Fmin are intertwined with IFF and ATR. For example, the region with abundant crossing fibers in the ATR in which the nerve fascicles were crossing the genu of the corpus callosum (Wiegell et al., 2000). Yet, we specifically focused on the communication of the corpus callosum reflected in Fmin and Fmaj in the DTI measures, in which Fmin represents the genu while Fmaj represents the splenium of the corpus callosum. The results showed a negative correlation with AHS in the FA measure in Fmin but not in Fmaj, suggesting that the genu of the corpus callosum plays a more important role in modulating analytic-holistic cognitive style.

### DTI Differences Between High vs. Low AHS Groups

To directly examine how the age differences in AHS are associated with DTI differences, we divided participants into two subgroups based on their AHS scores using the median-split method (AHS = 133). We found that the major difference in DTI between the two subgroups lies in FA and RD in the Fmin tract, suggesting that the difference in AHS score is highly correlated with Fmin. The inverse pattern between FA and RD in Fmin suggests that with increasing AHS, there is a general increase of omnidirectional diffusion (see Figure 11). This favors radial diffusivity (two minor directions of diffusion) over axial diffusivity, leading to a decrease in FA in Fmin. The results derived from the AHS group comparison strengthened the hypothesis that the corpus callosum is correlated with the holistic tendency in the elderly, especially the genu transmission.

### The Function of the Corpus Callosum Concerning Age-Related Differences in Analytic-Holistic Cognitive Style

How is the genu of the corpus callosum involved in age-related differences in holistic cognitive style? According to our findings, a robust association between the genu of the corpus callosum and AHS scores observed indicated the interhemispheric connectivity of the frontal cortex associated with age-related differences of analytic-holistic cognitive style tendencies. Previous research has found that the genu of the corpus callosum is involved in integrative (cooperative) function, whereas the splenium part is involved in inhibition function (Müller-Oehring et al., 2007). Hence, the relationship between lower FA in Fmin and higher AHS scores observed here seems to be consistent with the view of hemispheric integration (Banich and Belger, 1990; Banich et al., 1990).

According to the integrative (cooperative) hypothesis of the corpus callosum, we might expect to observe that the FA in the corpus callosum is decreased and RD is increased as a function of AHS scores. This would arise from the lower transmission between the two hemispheres (indicated by lower FA in corpus callosum), which might reduce cross-hemisphere processing. Thus, paradoxically, the advantage of within-hemisphere processing of the global (holistic) information processing (right hemisphere biased) would be enhanced.

Notably, we are aware that previous research has proposed at least three functional roles of the corpus callosum: hemispheric integration, insulation, and inhibition (Daselaar and Cabeza, 2005). In this study, we specifically focused on hemispheric integration and inhibition functions. Therefore, there might be alternative hypotheses regarding the interpretation of decreased FA in Fmin associated with higher AHS scores in the elderly. The corpus callosum may be involved in inhibition function, such that when one hemisphere is more strongly activated, it would suppress the orienting tendency of the other hemisphere. Under these circumstances, the age-related decrease in FA in Fmin seen here might reflect less inhibitory signals from the left-hemispheric processing to the right-hemispheric processing, which relatively enhances the global processing tendency for the elderly.

Likewise, according to the hemispheric insulation hypothesis, the decreased FA in Fmin might be related to the minimization of the communication between the two hemispheres, thus paradoxically enhancing the right-hemisphere processing that increases holistic processing tendency for older people. Nevertheless, the current study was not designed to directly disentangle these possible functional roles of the corpus callosum. Future studies incorporating functional MRI data are needed to systematically examine these alternative interpretations.

To conclude, this study can be considered as a pioneer study to demonstrate the complex relationships among age, cognitive style, and white matter integrity of the corpus callosum. The current findings help us to understand how age-related changes in holistic thinking tendencies may be related to the age-related differences in the white matter integrity, especially in the efficacy of cross-hemisphere transmission.

## Data Availability Statement

All datasets generated for this study are included in the article/Supplementary Material. The data that support the findings of this study are available from the corresponding author, SH (psyhsl@mail.ncku.edu.tw), upon request.

## Ethics Statement

The studies involving human participants were reviewed and approved by the Human Research Ethics Committee of the National Cheng Kung University, Tainan, Taiwan. The patients/participants provided their written informed consent to participate in this study.

## Author Contributions

SH: conceptualization, methodology, data curation, supervision, writing—original draft preparation. M-HY: data collection and analysis. Z-FY: data collection and analysis, writing—reviewing and editing. C-TY and C-HW: writing—reviewing and editing.

## Conflict of Interest

The authors declare that the research was conducted in the absence of any commercial or financial relationships that could be construed as a potential conflict of interest.

## Footnotes

^1^https://www.nitrc.org/projects/mricron/

^2^www.fmrib.ox.ac.uk/fsl

^3^https://fsl.fmrib.ox.ac.uk/fsl/fslwiki/Randomise

## References

[B1] Abe O. Aoki S. Hayashi N. Yamada H. Kunimatsu A. Mori H.. (2002). Normal aging in the central nervous system: quantitative MR diffusion-tensor analysis. Neurobiol. Aging 23, 433–441. 10.1016/s0197-4580(01)00318-9 11959406

[B2] Alexander A. L. Lee J. E. Lazar M. Field A. S. (2007). Diffusion tensor imaging of the brain. Neurotherapeutics 4, 316–329. 10.1016/j.nurt.2007.05.011 17599699 PMC2041910

[B3] Banich M. T. Belger A. (1990). Interhemispheric interaction: how do the hemispheres divide and conquer a task? Cortex 26, 77–94. 10.1016/s0010-9452(13)80076-7 2354647

[B4] Banich M. T. Goering S. Stolar N. Belger A. (1990). Interhemispheric processing in left-a nd right- handers. Int. J. Neurosci. 54, 197–208. 10.3109/00207459008986636 2265970

[B5] Beck A. T. Steer R. A. Brown G. K. (1996). Beck depression inventory-II. San Antonio 78, 490–498.

[B6] Bloom J. S. Hynd G. W. (2005). The role of the corpus callosum in interhemispheric transfer of information: excitation or inhibition? Neuropsychol. Rev. 15, 59–71. 10.1007/s11065-005-6252-y 16211466

[B7] Boekel W. Hsieh S. (2018). Cross-sectional white matter microstructure differences in age and trait mindfulness. PLoS One 13:e0205718. 10.1371/journal.pone.0205718 30321218 PMC6188777

[B8] Cabeza R. Anderson N. D. Locantore J. K. McIntosh A. R. (2002). Aging gracefully: compensatory brain activity in high-performing older adults. NeuroImage 17, 1394–1402. 10.1006/nimg.2002.1280 12414279

[B9] Chan C. Y. Chan A. B. Lee T. M. Hsiao J. H. (2018). Eye-movement patterns in face recognition are associated with cognitive decline in older adults. Psychon. Bull. Rev. 25, 2200–2207. 10.3758/s13423-017-1419-0 29313315

[B10] Chechlacz M. Mantini D. Gillebert C. R. Humphreys G. W. (2015). Asymmetrical white matter networks for attending to global versus local features. Cortex 72, 54–64. 10.1016/j.cortex.2015.01.022 25727548 PMC4643681

[B11] Chiarello C. Maxfield L. (1996). Varieties of interhemispheric inhibition, or how to keep a good hemisphere down. Brain Cogn. 30, 81–108. 10.1006/brcg.1996.0006 8811982

[B12] Choi I. Koo M. Choi J. A. (2007). Individual differences in analytic versus holistic thinking. Pers. Soc. Psychol. Bull. 33, 691–705. 10.1177/0146167206298568 17440200

[B13] Coffield F. Moseley D. Hall E. Ecclestone K. (2004). Should We be Using Learning Styles? What Research has to Say to Practice. London: Learning and Skills Research Centre.

[B14] Daselaar S. Cabeza R. (2005). “Age-related changes in hemispheric organization,” in Cognitive Neuroscience of Aging: Linking Cognitive and Cerebral Aging, eds Cabeza R. Nyberg L. Park D. C. (New York, NY: Oxford University Press), 325–353.

[B59] Demick J. Wapner S. (1991). “Field dependence-independence in adult development and aging,” in Cognitive style across the Life Span, eds Wapner S. Demick J. (Hillsdale, NJ: Lawrence Erlbaum Associates, Inc.), 245–268.

[B15] Dror I. E. Schmitz-Williams I. C. Smith W. (2005). Older adults use mental representations that reduce cognitive load: mental rotation utilizes holistic representations and processing. Exp. Aging Res. 31, 409–420. 10.1080/03610730500206725 16147460

[B16] Gazzaniga M. S. (2005). Forty-five years of split-brain research and still going strong. Nat. Rev. Neurosci. 6, 653–659. 10.1038/nrn1723 16062172

[B17] Hinkley L. B. N. Marco E. J. Findlay A. M. Honma S. Jeremy R. J. Strominger Z.. (2012). The role of corpus callosum development in functional connectivity and cognitive processing. PLoS One 7:e39804. 10.1371/journal.pone.0039804 22870191 PMC3411722

[B18] Hopper K. D. Patel S. Cann T. S. Wilcox T. Schaeffer J. M. (1994). The relationship of age, gender, handedness, and sidedness to the size of the corpus callosum. Acad. Radiol. 1, 243–248. 10.1016/s1076-6332(05)80723-8 9419493

[B19] Horsfield M. A. Jones D. K. (2002). Applications of diffusion-weighted and diffusion tensor MRI to white matter diseases-a review. NMR Biomed. 15, 570–577. 10.1002/nbm.787 12489103

[B20] Hsieh S. Yu Y. T. Chen E. H. Yang C. T. Wang C. H. (2020). ERP correlates of a flanker task with varying levels of analytic-holistic cognitive style. Pers. Individ. Diff. 153:109673 10.1016/j.paid.2019.109673

[B21] Jen C. H. Lien Y. W. (2010). What is the source of cultural differences?—Examining the influence of thinking style on the attribution process. Acta Psychol. 133, 154–162. 10.1016/j.actpsy.2009.10.011 19945090

[B23] Jenkinson M. Beckmann C. F. Behrens T. E. Woolrich M. W. Smith S. M. (2012). FSL. NeuroImage 62, 782–790. 10.1016/j.neuroimage.2011.09.015 21979382

[B22] Jenkinson M. Smith S. (2001). A global optimisation method for robust affine registration of brain images. Med. Image Anal. 5, 143–156. 10.1016/s1361-8415(01)00036-6 11516708

[B24] Jolly T. A. D. Cooper P. S. Wan Ahmadul Badwi S. A. Phillips N. A. Rennie J. L. Levi C. R.. (2016). Microstructural white matter changes mediate age-related cognitive decline on the Montreal Cognitive Assessment (MoCA). Psychophysiology 53, 258–267. 10.1111/psyp.12565 26511789

[B25] Junle Y. Youmin G. Yanjun G. Mingyue M. Qiujuan Z. Min X. (2008). A MRI quantitative study of corpus callosum in normal adults. J. Med. Coll. PLA 23, 346–351. 10.1016/s1000-1948(09)60005-8

[B26] Kozhevnikov M. (2007). Cognitive styles in the context of modern psychology: toward an integrated framework of cognitive style. Psychol. Bull. 133, 464–481. 10.1037/0033-2909.133.3.464 17469987

[B27] Lachman M. E. (2006). Perceived control over aging-related declines: adaptive beliefs and behaviors. Curr. Dir. Psychol. Sci. 15, 282–286. 10.1111/j.1467-8721.2006.00453.x

[B28] Leonard D. Straus S. (1997). Identifying how we think: the myers-briggs type indicator and the hermann brain dominance instrument. Harv. Bus. Rev. 75, 114–115.

[B29] Mealor A. D. Simner J. Rothen N. Carmichael D. A. Ward J. (2016). Different dimensions of cognitive style in typical and atypical cognition: new evidence and a new measurement tool. PLoS One 11:e0155483. 10.1371/journal.pone.0155483 27191169 PMC4871558

[B30] Meng Q. Wang B. Cui D. Liu N. Huang Y. Chen L.. (2019). Age-related changes in local and global visual perception. J. Vis. 19:10. 10.1167/19.1.10 30650433

[B31] Messick S. (1984). The nature of cognitive styles: problems and promise in educational practice. Educ. Psychol. 19, 59–74. 10.1080/00461528409529283

[B32] Miller A. (1987). Cognitive styles: an integrated model. Educ. Psychol. 7, 251–268. 10.1080/0144341870070401

[B33] Müller-Oehring E. M. Schulte T. Fama R. Pfefferbaum A. Sullivan E. V. (2009). Global-local interference is related to callosal compromise in alcoholism: a behavior-DTI association study. Alcohol. Clin. Exp. Res. 33, 477–489. 10.1111/j.1530-0277.2008.00858.x 19120053 PMC2651990

[B34] Müller-Oehring E. M. Schulte T. Raassi C. Pfefferbaum A. Sullivan E. V. (2007). Local-global interference is modulated by age, sex and anterior corpus callosum size. Brain Res. 1142, 189–205. 10.1016/j.brainres.2007.01.062 17335783 PMC1876662

[B35] Nash S. Rode G. Charnallet A. (2015). Global/local integration and corpus callosum: anatomical and behavioural study of case of Allgrove syndrome (triple-A syndrome). Ann. Phys. Rehabil. Med. 58:e30 10.1016/j.rehab.2015.07.077

[B36] Nasreddine Z. S. Phillips N. A. Bédirian V. Charbonneau S. Whitehead V. Collin I.. (2005). The montreal cognitive assessment, MoCA: a brief screening tool for mild cognitive impairment. J. Am. Geriatr. Soc. 53, 695–699. 10.1111/j.1532-5415.2005.53221.x 15817019

[B37] Navon D. (1977). Forest before trees: the precedence of global features in visual perception. Cogn. Psychol. 9, 353–383. 10.1016/0010-0285(77)90012-3

[B38] Nickerson R. S. perkins D. N. Smith E. E. (1985). The Teaching of Thinking. Hillsdale, NJ.: Lawrence Erlbaum Associates.

[B40] Oldfield R. C. (1971). The assessment and analysis of handedness: the Edinburgh inventory. Neuropsychologia 9, 97–113. 10.1016/0028-3932(71)90067-4 5146491

[B41] Pask G. (1972). A fresh look at cognition and the individual. Int. J. Man Machine Stud. 4, 211–216. 10.1016/s0020-7373(72)80002-6

[B42] Persson J. Nyberg L. Lind J. Larsson A. Nilsson L. G. Ingvar M.. (2006). Structure-function correlates of cognitive decline in aging. Cereb. Cortex 16, 907–915. 10.1093/cercor/bhj036 16162855

[B43] Peters M. (2004). The parallel brain: the cognitive neuroscience of the corpus callosum. JAMA J. Am. Med. Assoc. 291, 1006–1007. 10.1001/jama.291.8.1006

[B44] Prevedi G. P. Carli M. (1987). Adaption-innovation typology and right-left hemispheric preferences. Pers. Individ. Diff. 8, 681–686. 10.1016/0191-8869(87)90066-3

[B45] Racine C. A. Barch D. M. Braver T. S. Noelle D. C. (2006). The effect of age on rule-based category learning. Aging Neuropsychol. Cogn. 13, 411–434. 10.1080/13825580600574377 16887781

[B46] Radvansky G. A. (1999). Aging, memory, and comprehension. Curr. Dir. Psychol. Sci. 8, 49–53. 10.1111/1467-8721.00012

[B47] Riding R. J. Glass A. Douglas G. (1993). Individual differences in thinking: cognitive and neurophysiological perspectives. Educ. Psychol. 13, 267–279. 10.1080/0144341930130305

[B48] Roux F. Ceccaldi M. (2001). Does aging affect the allocation of visual attention in global and local information processing? Brain Cogn. 46, 383–396. 10.1006/brcg.2001.1296 11487288

[B49] Salat D. Ward A. Kaye J. A. Janowsky J. S. (1997). Sex differences in the corpus callosum with aging. Neurobiol. Aging 18, 191–197. 10.1016/s0197-4580(97)00014-6 9258896

[B50] Schulte T. Müller-Oehring E. M. (2010). Contribution of callosal connections to the interhemispheric integration of visuomotor and cognitive processes. Neuropsychol. Rev. 20, 174–190. 10.1007/s11065-010-9130-1 20411431 PMC3442602

[B51] Schulte T. Pfefferbaum A. Sullivan E. V. (2004). Parallel interhemispheric processing in aging and alcoholism: relation to corpus callosum size. Neuropsychologia 42, 257–271. 10.1016/s0028-3932(03)00155-6 14644111

[B52] Smith S. M. (2002). Fast robust automated brain extraction. Hum. Brain Mapp. 17, 143–155. 10.1002/hbm.10062 12391568 PMC6871816

[B53] Smith S. M. Jenkinson M. Johansen-Berg H. Rueckert D. Nichols T. E. Mackay C. E.. (2006). Tract-based spatial statistics: voxelwise analysis of multi-subject diffusion data. NeuroImage 31, 1487–1505. 10.1016/j.neuroimage.2006.02.024 16624579

[B54] Smith S. M. Jenkinson M. Woolrich M. W. Beckmann C. F. Behrens T. E. Johansen-Berg H.. (2004). Advances in functional and structural MR image analysis and implementation as FSL. NeuroImage 23, S208–S219. 10.1016/j.neuroimage.2004.07.051 15501092

[B55] Soares J. Marques P. Alves V. Sousa N. (2013). A hitchhiker’s guide to diffusion tensor imaging. Front. Neurosci. 7:31. 10.3389/fnins.2013.00031 23486659 PMC3594764

[B56] Sullivan E. V. Pfefferbaum A. Adalsteinsson E. Swan G. E. Carmelli D. (2002). Differential rates of regional brain change in callosal and ventricular size: a 4-year longitudinal MRI study of elderly men. Cereb. Cortex 12, 438–445. 10.1093/cercor/12.4.438 11884358

[B57] Sullivan E. V. Rohlfing T. Pfefferbaum A. (2010). Quantitative fiber tracking of lateral and interhemispheric white matter systems in normal aging: relations to timed performance. Neurobiol. Aging 31, 464–481. 10.1016/j.neurobiolaging.2008.04.007 18495300 PMC2815144

[B58] Vernooij M. W. de Groot M. van der Lugt A. Ikram M. A. Krestin G. P. Hofman A.. (2008). White matter atrophy and lesion formation explain the loss of structural integrity of white matter in aging. NeuroImage 43, 470–477. 10.1016/j.neuroimage.2008.07.052 18755279

[B60] Weis S. Kimbacher M. Wenger E. Neuhold A. (1993). Morphometric analysis of the corpus callosum using MR: correlation of measurements with aging in healthy individuals. Am. J. Neuroradiol. 14, 637–645. 8517352 PMC8333387

[B61] Wheeler-Kingshott C. A. Cercignani M. (2009). About “axial” and “radial” diffusivities. Magn. Reson. Med. 61, 1255–1260. 10.1002/mrm.21965 19253405

[B62] Wiegell M. R. Larsson H. B. Wedeen V. J. (2000). Fiber crossing in human brain depicted with diffusion tensor MR imaging. Radiology 217, 897–903. 10.1148/radiology.217.3.r00nv43897 11110960

[B63] Zhang X. Fung H. H. Stanley J. T. Isaacowitz D. M. Zhang Q. (2014). Thinking more holistically as we grow older? Results from different tasks in two cultures. Cult. Brain 2, 109–121. 10.1007/s40167-014-0018-4

