# Supplementary material for: Diffusion Tensor Imaging Revealing the Relation of Age-Related Differences in the Corpus Callosum With Cognitive Style
Source: Front Hum Neurosci. 2020 Jul 17;14:285. doi: 10.3389/fnhum.2020.00285 (PMC7379874; doi:10.3389/fnhum.2020.00285)
Supplement: Supplementary file 1 [file Table_1.DOCX]

**Supplementary Table 1. Correlations between age and fractional anisotropy (FA) in 18 white matter tracts**

|  | **r** | ***p*** | **BF_10_** | **BF_01_** |
| --- | --- | --- | --- | --- |
| **ATR_L** | -0.39 | 0.01 | 5.71 | 0.18 |
| **ATR_R** | -0.20 | 0.20 | 0.91 | 1.09 |
| **CG_L** | -0.54 | 0.00 | 71.43 | 0.01 |
| **CG_R** | -0.20 | 0.18 | 0.93 | 1.08 |
| **CH_L** | 0.29 | 0.06 | 1.87 | 0.57 |
| **CH_R** | 0.27 | 0.08 | 1.58 | 0.64 |
| **CST_L** | -0.20 | 0.20 | 0.90 | 1.11 |
| **CST_R** | -0.32 | 0.04 | 2.42 | 0.41 |
| **Fmaj** | -0.39 | 0.01 | 7.13 | 0.14 |
| **Fmin** | -0.78 | 3.77E-09 | 1.64E+06 | 6.11E-07 |
| **IFF_L** | -0.61 | 2.90E-05 | 604.80 | 0.00 |
| **IFF_R** | -0.57 | 1.01E-04 | 223.41 | 0.00 |
| **ILF_L** | -0.51 | 0.00 | 44.46 | 0.02 |
| **ILF_R** | -0.57 | 1.70E-04 | 142.30 | 0.01 |
| **SLF_L** | -0.49 | 0.00 | 29.77 | 0.03 |
| **SLF_R** | -0.46 | 0.00 | 20.41 | 0.05 |
| **UF_L** | -0.43 | 0.00 | 12.23 | 0.08 |
| **UF_R** | -0.37 | 0.02 | 5.00 | 0.20 |

**Supplementary Table 2. Correlations between age and mean diffusivity (MD) in 18 white matter tracts**

|  | **r** | ***p*** | **BF_10_** | **BF_01_** |
| --- | --- | --- | --- | --- |
| **ATR_L** | 0.34 | 0.02 | 4.18 | 0.24 |
| **ATR_R** | 0.40 | 0.01 | 9.40 | 0.11 |
| **CG_L** | 0.11 | 0.48 | 0.60 | 1.67 |
| **CG_R** | -0.20 | 0.19 | 0.93 | 1.08 |
| **CH_L** | -0.41 | 0.01 | 6.68 | 0.15 |
| **CH_R** | -0.21 | 0.19 | 0.96 | 1.04 |
| **CST_L** | -0.30 | 0.049 | 2.17 | 0.46 |
| **CST_R** | -0.20 | 0.20 | 0.91 | 1.10 |
| **Fmaj** | 0.08 | 0.65 | 0.50 | 1.99 |
| **Fmin** | 0.16 | 0.35 | 0.72 | 1.39 |
| **IFF_L** | 0.19 | 0.237 | 0.84 | 1.20 |
| **IFF_R** | 0.26 | 0.089 | 1.47 | 0.68 |
| **ILF_L** | 0.07 | 0.645 | 0.54 | 1.85 |
| **ILF_R** | 0.10 | 0.518 | 0.56 | 1.78 |
| **SLF_L** | 0.05 | 0.743 | 0.52 | 1.93 |
| **SLF_R** | 0.02 | 0.881 | 0.49 | 2.05 |
| **UF_L** | 0.05 | 0.762 | 0.50 | 2.00 |
| **UF_R** | 0.03 | 0.846 | 0.49 | 2.03 |

**Supplementary Table 3. Correlations between age and radial diffusivity (RD) in 18 white matter tracts**

|  | **r** | ***p*** | **BF_10_** | **BF_01_** |
| --- | --- | --- | --- | --- |
| **ATR_L** | 0.43 | 0.00 | 17.44 | 0.06 |
| **ATR_R** | 0.46 | 0.00 | 30.35 | 0.03 |
| **CG_L** | 0.44 | 0.01 | 11.55 | 0.09 |
| **CG_R** | 0.07 | 0.66 | 0.50 | 2.00 |
| **CH_L** | -0.38 | 0.02 | 4.79 | 0.21 |
| **CH_R** | -0.14 | 0.37 | 0.68 | 1.48 |
| **CST_L** | -0.05 | 0.78 | 0.51 | 1.98 |
| **CST_R** | 0.07 | 0.68 | 0.53 | 1.87 |
| **Fmaj** | 0.33 | 0.03 | 3.14 | 0.32 |
| **Fmin** | 0.52 | 0.00 | 54.98 | 0.02 |
| **IFF_L** | 0.40 | 0.01 | 7.33 | 0.14 |
| **IFF_R** | 0.41 | 0.01 | 8.98 | 0.11 |
| **ILF_L** | 0.32 | 0.05 | 2.32 | 0.43 |
| **ILF_R** | 0.32 | 0.04 | 2.60 | 0.38 |
| **SLF_L** | 0.20 | 0.21 | 0.91 | 1.10 |
| **SLF_R** | 0.18 | 0.25 | 0.83 | 1.21 |
| **UF_L** | 0.20 | 0.20 | 0.93 | 1.08 |
| **UF_R** | 0.19 | 0.23 | 0.87 | 1.16 |

**Table 4. Correlations between Analysis-Holism Scale (AHS) and fractional anisotropy (FA) in 18 tracts.**

|  | **r** | ***p*** | **BF_10_** | **BF_01_** |
| --- | --- | --- | --- | --- |
| **ATR_L** | -0.22 | 0.08 | 1.59 | 0.63 |
| **ATR_R** | -0.13 | 0.30 | 0.74 | 1.35 |
| **CG_L** | -0.19 | 0.14 | 1.14 | 0.88 |
| **CG_R** | -0.06 | 0.62 | 0.50 | 1.99 |
| **CH_L** | 0.13 | 0.32 | 0.72 | 1.40 |
| **CH_R** | 0.07 | 0.57 | 0.53 | 1.90 |
| **CST_L** | 0.07 | 0.56 | 0.54 | 1.87 |
| **CST_R** | -0.06 | 0.63 | 0.53 | 1.89 |
| **Fmaj** | -0.18 | 0.15 | 1.05 | 0.95 |
| **Fmin** | -0.36 | 0.00 | 21.26 | 0.05 |
| **IFF_L** | -0.29 | 0.02 | 4.55 | 0.22 |
| **IFF_R** | -0.28 | 0.02 | 3.87 | 0.26 |
| **ILF_L** | -0.28 | 0.03 | 3.19 | 0.31 |
| **ILF_R** | -0.29 | 0.02 | 3.81 | 0.26 |
| **SLF_L** | -0.24 | 0.05 | 2.09 | 0.48 |
| **SLF_R** | -0.17 | 0.18 | 0.97 | 1.03 |
| **UF_L** | -0.18 | 0.05 | 2.16 | 0.46 |
| **UF_R** | -0.17 | 0.31 | 0.72 | 1.40 |

**Table 5. Correlations between Analysis-Holism Scale (AHS) and mean diffusivity (MD) in 18 tracts.**

|  | **r** | ***p*** | **BF_10_** | **BF_01_** |
| --- | --- | --- | --- | --- |
| **ATR_L** | 0.31 | 0.01 | 8.99 | 0.11 |
| **ATR_R** | 0.33 | 0.01 | 10.96 | 0.09 |
| **CG_L** | 0.16 | 0.20 | 0.94 | 1.07 |
| **CG_R** | 0.06 | 0.61 | 0.53 | 1.88 |
| **CH_L** | 0.08 | 0.55 | 0.58 | 1.72 |
| **CH_R** | 0.07 | 0.57 | 0.56 | 1.80 |
| **CST_L** | -0.06 | 0.64 | 0.51 | 1.95 |
| **CST_R** | 0.02 | 0.90 | 0.49 | 2.06 |
| **Fmaj** | 0.00 | 0.99 | 0.46 | 2.20 |
| **Fmin** | 0.22 | 0.09 | 1.52 | 0.66 |
| **IFF_L** | 0.21 | 0.09 | 1.48 | 0.68 |
| **IFF_R** | 0.24 | 0.05 | 2.22 | 0.45 |
| **ILF_L** | 0.08 | 0.51 | 0.59 | 1.71 |
| **ILF_R** | 0.14 | 0.28 | 0.75 | 1.33 |
| **SLF_L** | 0.13 | 0.33 | 0.72 | 1.40 |
| **SLF_R** | 0.14 | 0.28 | 0.76 | 1.31 |
| **UF_L** | 0.21 | 0.09 | 1.48 | 0.67 |
| **UF_R** | 0.16 | 0.20 | 0.92 | 1.08 |

**Table 6. Correlations between Analysis-Holism Scale (AHS) and radial diffusivity (RD) in 18 tracts.**

|  | **r** | ***p*** | **BF_10_** | **BF_01_** |
| --- | --- | --- | --- | --- |
| **ATR_L** | 0.33 | 0.01 | 11.89 | 0.08 |
| **ATR_R** | 0.34 | 0.00 | 12.99 | 0.08 |
| **CG_L** | 0.23 | 0.07 | 1.75 | 0.57 |
| **CG_R** | 0.10 | 0.44 | 0.58 | 1.71 |
| **CH_L** | 0.03 | 0.85 | 0.51 | 1.95 |
| **CH_R** | 0.10 | 0.45 | 0.62 | 1.61 |
| **CST_L** | -0.07 | 0.58 | 0.55 | 1.81 |
| **CST_R** | 0.05 | 0.71 | 0.53 | 1.89 |
| **Fmaj** | 0.13 | 0.27 | 0.72 | 1.40 |
| **Fmin** | 0.32 | 0.01 | 7.37 | 0.14 |
| **IFF_L** | 0.28 | 0.02 | 3.55 | 0.28 |
| **IFF_R** | 0.28 | 0.02 | 3.87 | 0.26 |
| **ILF_L** | 0.20 | 0.12 | 1.29 | 0.77 |
| **ILF_R** | 0.22 | 0.08 | 1.60 | 0.63 |
| **SLF_L** | 0.18 | 0.16 | 1.07 | 0.94 |
| **SLF_R** | 0.15 | 0.24 | 0.85 | 1.18 |
| **UF_L** | 0.22 | 0.09 | 1.53 | 0.65 |
| **UF_R** | 0.19 | 0.13 | 1.21 | 0.83 |

Abbreviations: ATR = anterior thalamic radiation; CG = cingulum; CH = cingulum of the hippocampus; CST = corticospinal tract; Fmaj = corpus callosum forceps major; Fmin = corpus callosum forceps minor; IFF = inferior fronto-occipital fasiculus; ILF = inferior longitudinal fasiculus; SLF = superior longitudinal fasiculus; UF = uncinate fasiculus.
